# Strain background of Candida albicans interacts with SIR2 to alter phenotypic switching

**DOI:** 10.1099/mic.0.001444

**Published:** 2024-03-06

**Authors:** Andrew L. Woodruff, Judith Berman, Matthew Anderson

**Affiliations:** 1 Department of Microbiology, The Ohio State University, Columbus, OH, 43210, USA; 2 Shmunis School of Biomedical and Cancer Research, The George S Wise Faculty of Life Sciences, Tel Aviv University, Tel Aviv, 69978, Israel; 3 Department of Microbial Infection and Immunity, The Ohio State University, Columbus, OH, 43210, USA; 4 Department of Medical Genetics, Laboratory of Genetics, University of Wisconsin – Madison, Madison, WI, 53706, USA; 5 Center for Genomic Science Innovation, University of Wisconsin – Madison, Madison, WI, 53706, USA

**Keywords:** aneuploidy, *Candida*, white opaque switching, epigenetics

## Abstract

The genetic background between strains of a single species and within a single strain lineage can significantly impact the expression of biological traits. This genetic variation may also reshape epigenetic mechanisms of cell identity and environmental responses that are controlled by interconnected transcriptional networks and chromatin-modifying enzymes. Histone deacetylases, including sirtuins, are critical regulators of chromatin state and have been directly implicated in governing the phenotypic transition between the ‘sterile’ white state and the mating-competent opaque state in *Candida albicans,* a common fungal commensal and pathogen of humans. Here, we found that a previously ambiguous role for the sirtuin *SIR2* in *C. albicans* phenotypic switching is likely linked to the genetic background of mutant strains produced in the RM lineage of SC5314. *SIR2* mutants in a specific lineage of BWP17 displayed increased frequencies of switching to the opaque state compared to the wild-type. Loss of *SIR2* in other SC5314-derived backgrounds, including newly constructed BWP17 *sir2*Δ/Δ mutants, failed to recapitulate the increased white–opaque switching frequencies observed in the original BWP17 *sir2*Δ/Δ mutant background. Whole-genome sequencing revealed the presence of multiple imbalanced chromosomes and large loss of heterozygosity tracts that likely interact with *SIR2* to increase phenotypic switching in this BWP17 *sir2*Δ/Δ mutant lineage. These genomic changes are not found in other SC5314-derived *sir2*Δ/Δ mutants that do not display increased opaque cell formation. Thus, complex karyotypes can emerge during strain construction that modify mutant phenotypes and highlight the importance of validating strain background when interpreting phenotypes.

## Introduction

Distinct lineages of the same species often display significant phenotypic diversity that can be caused by underlying genetic variation. Major sources of this variation in mitotically growing heterozygous diploids include loss of heterozygosity (LOH), copy number variations (CNVs) and point mutations that can directly alter key effector genes or indirectly modulate their function through remodelled regulatory or genetic networks. The significance of genetic background is acutely illustrated in comparative mutant screens that use multiple strain backgrounds. For example, pairwise comparison of single-gene-deletion mutants constructed in multiple backgrounds of *Caenorhabditis elegans* or *Saccharomyces cerevisiae* found different phenotypes for ~20 % of all genes in the genome [1,3]. Transcriptional regulators and chromatin-modifying enzymes often play a role in these phenotypic differences, presumably because of their pleiotropic roles in gene expression [2,4, 5].

Seventeen major clades define the species architecture of the common commensal and opportunistic human fungal pathogen *Candida albicans* and reflect the accumulation of inherited mutations during its primarily asexual evolution [6]. Despite *C. albicans* strains exhibiting up to 2 % nucleotide divergence, the vast majority of molecular investigations have used a single genetic background, SC5314, which serves as the genome reference strain [7,10]. Much of this work was facilitated by construction of auxotrophic mutants in the SC5314 background that allowed the development of tools for molecular manipulation of this diploid, largely asexual species [11]. Gene deletions, replacements, complementation and overexpression have led to important insights into the genetic basis of traits required for *C. albicans* virulence [11,12]. Two related auxotrophic lineages (series of strains derived by sequential molecular modifications) were built in the SC5314 background for common laboratory use: the RM lineage and the SN lineage. From the RM lineage, strain BWP17 carries three auxotrophies (*arg4*Δ/Δ*, his1*Δ/Δ and *ura3*Δ/Δ) and was a workhorse for complementation-based selection during strain construction to investigate a range of biological processes [12,20]. With each successive step in construction, RM isolates accumulated different LOH and CNV events [21], presumably through the process of DNA transformation that increases the gain and loss of imbalanced chromosomes [22,23]. Strains in the RM lineage contain an inadvertent disruption of *IRO1,* which is adjacent to the *URA3* gene [24], and loss of 40 kilobases (kb) of the right arm of chromosome 5 (Chr5R) just distal to *HIS1* and extending to the telomere [25]. Loss of *URA3* and/or *IRO1+URA3* deletions, present in the RM lineage, reduce virulence in *C. albicans* [26,28]. The SN lineage is derived from the RM lineage but avoided the truncation event on Chr5R and restored one intact copy of *IRO1* and *URA3* [29], thereby repairing some of the defects in RM strains.

The same defined mutation can produce different phenotypic outcomes across *C. albicans* backgrounds. Large karyotypic changes, such as Chr4 trisomy, can promote antifungal drug resistance in some strains but have no effect in others [30]. Furthermore, genetic backgrounds respond differently to targeted deletion of central transcriptional regulators of biofilm formation [4,31]. For example, *bcr1*Δ/Δ cells in the P57055 strain fail to form biofilms, whereas deletion of *BCR1* in SC5314 minimally alters biofilm mass, biofilm architecture and transition between yeast and hyphal cell states. Similarly, genetically distinct lineages in the same genetic background can produce different phenotypes, but nearly all examples are restricted to comparison between diploid genotypes and their aneuploid variants [32,34].

Interconversion between the ‘sterile’ white and mating-competent opaque cell states is a well-characterized phenotypic switch in * C. albicans* [35,38]. Morphologically, white cells are round or ovoid and form smooth domed colonies, whereas opaque cells are elongated, often appearing club shaped, and form flatter, dull colonies. Approximately 20 % of the genome is expressed differently between the white and opaque states [39], leading to major differences in metabolism [40,41], filamentation programmes [42,43] and mating responses [38,44]. Both cell states are capable of dissemination to host organs in a murine model of infection but differ in their relative colonization among organ systems [45,46]. Opaque cells were also found to be phagocytosed less efficiently by macrophage than white cells and may therefore more efficiently avoid destruction by innate immune cells [47,49].

Access to the opaque state facilitates entering an alternative mating system termed parasex. The first requirement for formation of opaque cells is mating type homozygosity, which can be achieved via LOH or targeted disruption of one of two idiomorphs at the mating type-like (*MTL*) locus – either *MTL***a** or *MTL*α – to produce homozygous or hemizygous cells, respectively [38]. *MTL* homozygous cells can switch to the opaque state via a low frequency epigenetic event that occurs every 1 in ~10 000 cell divisions under ambient growth conditions [50,51]. The newly acquired opaque cell state is heritable but can revert to the white state via similarly stochastic cell state transitions [37,52]. Opaque cells are able to undergo pheromone-induced polarized growth, or ‘shmooing’, towards opaque cells of the opposing mating type and initiate a process of cell–cell fusion and karyogamy to produce tetraploid mating products that can be induced to undergo a process of uncoordinated ploidy reduction called concerted chromosome loss [44,53, 54].

Epigenetic switching between the white and opaque cell states is governed by a network of chromatin-modifying enzymes and transcriptional feedback loops. A class of NAD^+^-dependent histone deacetylases referred to as sirtuins are central to white–opaque switching but do not show universal preference for promoting either cell state [55,56]. Some sirtuins (e.g. *HST2* and *HST3*) destabilize the white state or favour opaque stability, whereas *HST1* destabilizes the opaque state. Altered phenotypic switching due to sirtuin mutations is dependent on *MTL* homozygosity and does not bypass the *MTL***a**1*–MTL*α2 heterodimer expressed in the *MTL***a**/α background [57]. The first investigation of the *C. albicans* sirtuin *SIR2* used a *sir2*Δ/Δ isolate in the CAI4 background, a predecessor to the RM lineage*,* and reported an increase in colony variation and phenotypic switching away from the yeast morphology of white cells but the researchers were later unable to replicate their results [58]. Conversely, a broader survey of histone modifiers for their role in white–opaque switching conducted in the SN lineage did not detect any phenotypes associated with loss of *SIR2* [55]. The reason for this difference in phenotypic switching in *sir2*Δ/Δ mutants is not clear.

Here, we compared the function of *SIR2* in white–opaque phenotypic switching between multiple strains all derived from SC5314: BWP17 (RM lineage), SN152 (SN lineage) and new auxotrophic strains directly constructed in SC5314 using CRISPR/Cas9. Disruption of *SIR2* in an initial set of BWP17 mutants increased switching to the opaque state and was partially restored by complementation with a wild-type *SIR2* allele, whereas *sir2*Δ/Δ strains in the SN lineage, a prototrophic SC5314 strain, several reconstructed BWP17 backgrounds and a CRISPR-competent BWP17 strain set displayed wild-type frequencies of white–opaque switching. Tests of previously defined molecular functions of *SIR2* did not reveal any differences between these strains. Nor were differences in white–opaque switching due to reduced growth rates of the original BWP17 *sir2*Δ/Δ mutants or gene deletions used to construct auxotrophic markers in BWP17. Instead, increased white–opaque switching appears to be associated with complex karyotypic changes in the original set of BWP17-derived *sir2*Δ/Δ mutants, suggesting an interaction between gene or allelic dosage on specific chromosomes and *SIR2*-mediated regulation of cell identity. These results reinforce the importance of determining strain genotypes during molecular characterization to avoid misinterpretations of experimental results.

## Methods

### Media and reagents

Yeast extract–peptone–dextrose (YPD) and synthetic complete dextrose (SCD) media were prepared as previously described [59]. Yeast extract–peptone–maltose (YPM) medium was prepared as YPD but used maltose as the carbon source in place of glucose. YPD and SCD containing 200 µg ml^−1^ nourseothricin (Werner Bioagents, Jena, Germany) were used to select for nourseothricin-resistant (NAT^R^) strains. SCD lacking l-leucine (SCD-LEU), l-arginine (SCD-ARG), l-histidine (SCD-HIS), or uracil and uridine (SCD-URA) were used to select for auxotrophic strains.

### Strain and plasmid construction

The strains, oligonucleotides (oligos) and plasmids used in this work are provided in Tables S1–S3 (available in the online version of this article), respectively. For the SC5314-derived strain set, CRISPR-mediated deletion of *SIR2*, *URA3*, *ARG4*, *HIS1* and *IRO1* was performed with the oligos listed in Table S2 as previously described using a modified lithium acetate transformation protocol [60]. Loss of targeted loci was confirmed by polymerase chain reaction (PCR) with oligos that amplify target genes [open reading frame (ORF) Chk] and by using phenotypic assays (for auxotrophies). For the CRISPR-competent BWP17 strains, CRISPR-mediated deletion of *SIR2* was performed using the same oligos as the SC5314-derived strain set.

To generate *LEU2* heterozygous (CRISPR-competent) BWP17 strains, we used plasmid p1 to delete one copy of *LEU2*. After PCR amplification of the deletion cassette and ethanol precipitation, the cassette was integrated into *C. albicans* as previously described using a standard lithium acetate transformation [60]. After verification of *LEU2* heterozygosity via PCR for the integration flanks and ORF checks, *LEU2* heterozygous strains had the *SAT1-FLP* cassette recycled by plating to ~100 colonies on solid YPM medium top-spread with either 10 or 20 µg ml^−1^ NAT. Small colonies (indicative of loss of *SAT1*) were then patched to YPD with or without 200 µg ml^−1^ NAT to screen for NAT-sensitive (NAT^S^) strains. Strains that were NAT^S^ were reverified for *LEU2* heterozygosity once more via PCR with ORF and Up/Dwn (produce a smaller amplicon if at least one allele is deleted) checks.

To generate *MTL* hemizygous strains, we used plasmids p39 and p40 to delete the *MTL***a** or *MTL*α loci, respectively [61]. After PCR amplification of deletion cassettes and ethanol precipitation, the cassettes were integrated into *C. albicans* as previously described using a standard lithium acetate transformation [62]. After verification of *MTL* genotype via PCR (*MTL***a**: oligos 73+74; *MTL*α: oligos 75+76), *MTL* hemizygous strains had the *SAT1-FLP* cassette recycled as described above.

For single deletions of *URA3*, *ARG4* and *HIS1*, colonies were screened for non-functionality of genes by first selecting for NAT^R^ transformant colonies on solid YPD+NAT medium, followed by replica plating to NAT-containing solid SCD-URA, SCD-ARG and SCD-HIS media, respectively. Colonies that were unable to grow on the solid drop-out media were individually patched to solid YPD+NAT, and then replica-plated to the respective drop-out medium to verify that the patches were correctly identified as being auxotrophic for the marker. After excision of the CRISPR cassette on SCD-LEU and verification that strains were LEU^+^/NAT^S^ (indicative of excision of the CRISPR cassette from the *LEU2* locus), they were then patched to all three solid drop-out media to verify that only one of the genes lacked functionality.

To generate triple auxotrophic strains for uridine, arginine and histidine, we used the single-deletion strains generated above and targeted the remaining two genes to be deleted simultaneously by using paired guide RNA–donor DNA (gRNA–dDNA) sets. Colonies were screened for gene loss in the same manner as described above but were replica-plated to both NAT-containing solid drop-out media corresponding to the two new deletions. After excision of the CRISPR cassette, strains were patched to all three solid drop-out media to verify that all three genes were absent. Following deletion of *SIR2* in these strains as described above, loss of all four target genes was verified via PCR (ORF Chk) to support the observed absence of functionality. For triple auxotrophic strains that were *sir2*Δ/Δ, we generated *IRO1* deletion strains as described above for CRISPR-mediated single-gene deletions. Both full coding sequence *IRO1* deletions and 3′-*IRO1* deletions were performed to generate two sets of strains that harboured clean deletions of *IRO1* or deletions of *IRO1* that mimicked the unintentional deletion of the 3′ end of *IRO1* in the BWP17 lineage, respectively.

Construction of the *SIR2* complementation plasmid p27 was performed by cloning PCR-amplified *SIR2* from crushed SC5314 cells (including the promoter, coding sequence and downstream) into pSFS2A using restriction enzymes ApaI and XhoI. Construction of a second *SIR2* complementation plasmid, p73, was performed coincidently using gap-repair cloning as described elsewhere [63]. Briefly, *SIR2* was PCR-amplified from SC5314 genomic DNA (including the promoter, coding sequence and downstream) with oligos encoding 20 bp ends homologous to pSFS2A, and pSFS2A was linearized via PCR amplification with oligos containing 20 bp of homology to *SIR2*, generating 40 bp of total overlap on each end of both PCR fragments. After digestion of residual plasmid template using DpnI, each PCR product was gel purified and transformed into chemically competent DH5α to be assembled into an intact plasmid. Both plasmids were Sanger sequenced. Plasmid p73 was identical to the *C. albicans* Assembly 21 sequence, whereas p27 contained three variants, two of which produced missense changes in Sir2 at sites not connected to known functions. To complement plasmid-borne *SIR2* into the *sir2*Δ/Δ strains, the plasmids were linearized in the *SIR2* promoter using MluI and transformed into *C. albicans*. After verification that *SIR2* integrated into the correct genomic location via PCR for the upstream integration flank and ORF checks, *SIR2*-complemented strains had the *SAT1-FLP* cassette excised as described above.

### White-to-opaque switching assays

White-to-opaque switching frequency was determined in a manner similar to previously described methods. Briefly, cells were struck onto solid SCD medium from glycerol stocks and were grown at room temperature for 4 to 5 days. After growth, three to six colonies that were pure white populations upon visual inspection were resuspended in 1× phosphate-buffered saline (PBS) and plated onto solid SCD medium at approximately 100–125 cells per plate. Following incubation of plates for 7 days at room temperature, the switching frequency was calculated as the percentage of all colonies present that contained opaque sectors or were entirely opaque. Experiments for each genotype were performed with a minimum of three biological replicates, where each biological replicate was an independent resuspension of three to six colonies.

### Quantitative reverse transcription PCR (qRT-PCR)

Three to four independent cultures for each strain investigated were grown overnight at 30 °C in liquid YPD medium. The next day, cells were diluted 1 : 100 in fresh liquid YPD medium and were grown at 30 °C for 3 to 4 h. RNA was then harvested from the cultures using the MasterPure Yeast RNA Purification kit (Epicenter, Madison, WI, USA) according to the manufacturer’s instructions, and was treated with DNase I. cDNA was generated with 1 µg of the treated RNA using oligo(dT)_18_ and SuperScript III Reverse Transcriptase (Thermo Scientific, Waltham, MA, USA). The cDNA was screened for the presence of genomic DNA contamination via PCR using an intron-spanning primer set for ribosomal protein large subunit 6 (*RPL6*) listed in Table S2 [64], and clean cDNA was used for quantitative qRT-PCR. qRT-PCR was performed with PowerUp SYBR Green (Applied Biosystems, Foster City, CA, USA) using an Applied Biosystems QuantStudio 3 Real-Time PCR System with the oligos provided in Table S2. Gene expression was calculated using the 2^−ΔCt^ method, where expression of each gene was normalized to *ACT1* expression. Experiments for each gene were performed with a minimum of four biological replicates per genotype with two technical replicates each, where each biological replicate was an independent overnight culture.

### Growth curve assays

Overnight cultures were grown at 30 °C in a 96 deep-well plate, with shaking at 125 r.p.m. with 300 µl of liquid SCD medium. The following day, overnight cultures were diluted 1 : 40 into H_2_O and then 1 : 50 into fresh liquid SCD medium for a final volume of 150 µl in a clear Greiner CELLSTAR 96-well flat-bottom cell culture plate (Greiner Bio-One). The plate was then sealed with a sterile, optically transparent polyester adhesive sealing film. Optical density at 600 nm (OD_600_) was measured every 15 min for 48 h at 25–26 °C using a BioTek Synergy H1 microplate reader (BioTek Instruments, Winooski, VT, USA), with double orbital continuous shaking at fast orbital speed and a frequency of 425 c.p.m. (3 mm). The polynomial measurement of the curve was used to derive the maximum doubling time. Experiments for each genotype were performed with a minimum of four biological replicates with two technical replicates each, where each biological replicate was an independent overnight culture.

### Whole-genome sequencing

For MAY307, MAY314, MAY315, MAY1013 and MAY1244, DNA was extracted from overnight cultures grown at 30 °C (~10^8^ cells) using the Zymogen Quick-DNA Fungal/Bacterial Miniprep kit (Zymogen, cat# D6005). gDNA concentration was quantified using the Qubit dsDNA Broad Range Assay kit (Thermo Fisher, cat# Q32853). Following quantification, the samples were sent to the Applied Microbiology Services Laboratory (AMSL) at The Ohio State University for processing. Libraries were constructed via tagmentation and dual-index barcoding using a modified protocol for the Illumina (L) Tagmentation kit (Illumina, cat# 20040537) to produce average final fragment sizes of approximately 450–500 bp. The libraries were sequenced for 2×150 paired-end reads on an Illumina NextSeq 2000. Reads were demultiplexed and Illumina adaptors were trimmed by AMSL. Read quality was assessed using FastQC (v0.11.7) [65], and low-quality positions were trimmed using Trimmomatic (v0.35 LEADING:20 TRAILING:20 SLIDINGWINDOW:4 : 20 MINLEN:35) [66], after which the trimmed data were checked again using FastQC. Reads were mapped to the *C. albicans* reference genome Assembly 21 (A21-s02-m09-r10) – obtained 2 March 2021 from the *Candida* Genome Database website (http://www.candidagenome.org/download/sequence/C_albicans_SC5314/Assembly21/current/C_albicans_SC5314_A21_current_chromosomes.fasta.gz) – using Bowtie 2 (v2.2.6–2) with parameters ‘−3 1’ to improve downstream analysis [67]. Samtools (v0.1.19) was then used to generate .bam files, read sorting and sample indexing [68]. Read alignment quality was interrogated via visual scanning using Integrative Genomics Viewer (IGV, v2.9.2) for aneuploidy, LOH and major genomic rearrangements [69]. Secondary checks for ploidy and heterozygosity were performed using Y_MAP_ for visualization [70].

For all other sequenced strains (Fig. S3), strains were struck onto solid YPD medium and grown at 30 °C for 2 days, after which the strains were processed for sequencing by SeqCoast Genomics (Portsmouth, NH, USA). Briefly, DNA from each strain was extracted using the MagMAX Microbiome Ultra Nucleic Acid Isolation kit and prepared for whole-genome sequencing using the Illumina DNA Prep tagmentation kit and unique dual indexes. Sequencing was performed on the Illumina NextSeq 2000 platform to produce 2×150 bp paired-end reads. Reads were demultiplexed, trimmed and assessed using FastQC. Ploidy and heterozygosity were interrogated using Y_MAP_ [70].

### Data accessibility

The data sets generated and/or analysed during the current study are available from the corresponding author on reasonable request. All sequencing data are available through the National Center for Biotechnology Information (NCBI) accession PRJNA1020173. All unique materials are available upon request from the authors or from commercial sources.

## Results

An initial set of BWP17 *sir2*Δ/Δ strains (referred to as the ‘original BWP17 *SIR2* set’) exhibited phenotypes indicative of increased switching to the opaque state: in *MTL* homozygous lineages grown on SCD agar, up to 25 % of *MTL***a**/**a** and *MTL*α/α *sir2*Δ/Δ colonies had flattened, dull sectors composed of cells that appeared to be in the opaque state. This rate of spontaneous white–opaque (W/O) switching was significantly greater than the typical 1–5 % switching frequency reported in SC5314 *MTL* homozygous strains [52], suggesting a potential role for *SIR2* in phenotypic switching.

### Increased phenotypic switching in *sir2*Δ/Δ mutants is lineage-dependent

**Fig. 1.**
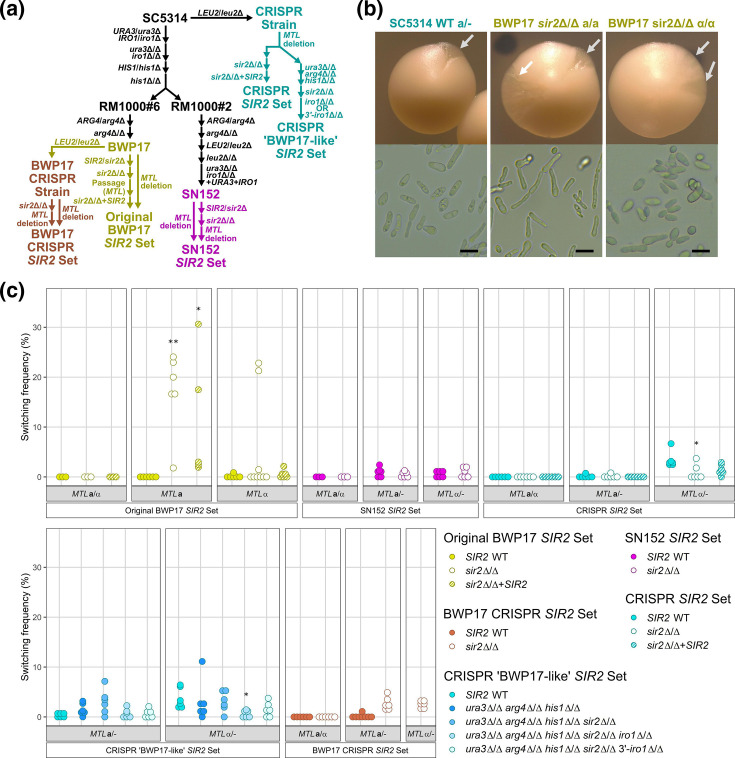
Strain-specific loss of *SIR2* increases white-to-opaque switching. (**a**) Construction diagram of *SIR2* mutants in the BWP17 (yellow for the original set, orange for the CRISPR-derived set), SN152 (magenta) and CRISPR-derived SC5314 (cyan) backgrounds. *MTL*, mating type-like locus. (**b**) Opaque sectors and the corresponding cells are displayed from wild-type and *sir2*Δ/Δ mutants grown on solid SCD medium at room temperature for 7 days. ‘**a**’ and ‘α‘ indicate the *MTL* genotype. Grey arrows indicate opaque sectors. Scale bars, 10 µm. (**c**) One hundred cells were plated from three to six pure white colonies onto solid SCD medium and grown at room temperature for 7 days and the frequency of opaque sectors or colonies was quantified. Each dot represents an independent switching assay for the indicated *MTL* and *SIR2* genotype and follows the same colouring scheme as the construction diagram. *n*≥3 biological replicates. *, *P*<0.05 (Kruskal–Wallis test with Dunn’s post-hoc against wild-type). **, *P*<0.01 (Kruskal–Wallis test with Dunn’s post-hoc against wild-type). WT, wild-type.

To comprehensively determine the effect of *SIR2* on white–opaque switching, we constructed *sir2*Δ/Δ mutants in multiple SC5314-derived backgrounds, including BWP17, SN152 and a CRISPR-competent prototrophic SC5314 strain. We also assayed *SIR2* in a newly rebuilt ‘BWP17-like’ strain in the CRISPR-competent SC5314 background, and a CRISPR-competent BWP17 strain. This strategy assayed the biological role of *SIR2* at multiple branch points and steps in the engineering of SC5314 for molecular biology (Fig. 1a). BWP17 was originally built from SC5314 to intentionally use complementation of the disrupted loci (*URA3*, *HIS1*, and *ARG4*) for selectable markers during strain construction. The CRISPR-competent SC5314 strain is entirely wild-type with the exception of a heterozygous *LEU2* locus used in recycling the Cas9 nuclease and guide RNA (gRNA) cassette [60]. Disruption of *URA3*, *HIS1*, *ARG4* and *IRO1* in this CRISPR background reproduced the BWP17 genetic background without the accumulation of LOH tracts and a Chr5R hemizygous truncation that arose during RM lineage construction [21]. Deletion of *SIR2* in each strain background can therefore distinguish between common or lineage-specific roles for *SIR2* in the regulation of white–opaque switching in the SC5314 lineage.

Only deletion of *SIR2* in the original BWP17 *SIR2 MTL* homozygous strain set altered rates of white–opaque switching, while deletion of *SIR2* in *MTL* hemizygous strains of the other strain sets had no effect. To measure phenotypic switching, cells of each genotype were plated for single colonies and allowed to grow at ambient temperature for 7 days on synthetic rich medium. Sectors of opaque cells in otherwise white colonies or entire opaque colonies arising spontaneously give an indication of W/O switching rates (Fig. 1b). As expected, no phenotypic switching occurred in *MTL***a**/α *sir2*Δ/Δ mutants from any of the backgrounds, including BWP17, due to repression of opaque-regulated genes by the *MTL***a**1*–MTL*α2 heterodimer [71] (Fig. 1c, Table 1). *MTL***a**/− and *MTL*α/− strains constructed by targeted loss of an *MTL* idiomorph that have both wild-type *SIR2* alleles gave rise to opaque sectors in 1–2 % of plated colonies on average in all strain backgrounds. Loss of *SIR2* in the original BWP17 *SIR2* set significantly increased phenotypic switching to as much as 25 % of colonies in both *MTL***a** and *MTL*α backgrounds [Fig. 1c, Kruskal–Wallis test (*MTL***a**, H(2) = 11.6, *P*=0.003; *MTL*α, H(2) = 1.2, *P*=0.556), Dunn’s post-hoc test (*MTL***a**, WT – *sir2*Δ/Δ, *P*=0.0048)]. By contrast, SN152 *sir2*Δ/Δ mutants, CRISPR-competent SC5314 *sir2*Δ/Δ mutants and the reconstructed ‘BWP17-like’ CRISPR-competent *sir2*Δ/Δ mutants formed opaque sectors or colonies at identical rates to their *SIR2* wild-type background (Fig. 1c, Table 1). Furthermore, generation of new BWP17 *MTL***a**/− *sir2*Δ/Δ mutants or new BWP17 *MTL*α/− *sir2*Δ/Δ mutants (labelled as ‘BWP17 CRISPR *SIR2* set’) failed to recapitulate the high-frequency switching phenotype observed in the original BWP17 *SIR2* set despite being constructed in the wild-type BWP17 background.

**Table 1.** White–opaque switching frequencies of *SIR2* strains

Background	*SIR2* genotype	*MTL* genotype*	Average switching frequency (%)†
	*SIR2*/*SIR2*	*MTL***a**/*MTL*α	0.00±0.00
		*MTL***a**/*MTL*αΔ::FRT	0.00±0.00
		*MTL***a**Δ::FRT/*MTL*α	0.14±0.35
	*sir2*Δ/*sir2*Δ	*MTL***a**/*MTL*α	0.00±0.00
BWP17 (original *SIR2* set)		*MTL***a**/*MTL***a**	16.46±6.60
		*MTL*α/*MTL*α	5.06±9.65
	*sir2*Δ/*sir2*Δ::*SIR2*	*MTL***a**/*MTL*α	0.00±0.00
		*MTL***a**/*MTL***a**	11.10±12.70
		*MTL*α/*MTL*α	0.55±0.83
SN152	*SIR2*/*SIR2*	*MTL***a**/*MTL*α	0.00±0.00
		*MTL***a**/*MTL*αΔ::FRT	0.99±0.91
		*MTL***a**Δ::FRT/*MTL*α	0.54±0.60
	*sir2*Δ/*sir2*Δ	*MTL***a**/*MTL*α	0.00±0.00
		*MTL***a**/*MTL*αΔ::FRT	0.49±0.56
		*MTL***a**Δ::FRT/*MTL*α	0.85±0.98
SC5314 *LEU2*/*leu2*Δ	*SIR2*/*SIR2*	*MTL***a**/*MTL*α	0.00±0.00
		*MTL***a**/*MTL*αΔ::FRT	0.24±0.35
		*MTL***a**Δ::FRT/*MTL*α	3.57±1.76
	*sir2*Δ/*sir2*Δ	*MTL***a**/*MTL*α	0.00±0.00
		*MTL***a**/*MTL*αΔ::FRT	0.13±0.33
		*MTL***a**Δ::FRT/*MTL*α	0.92±1.54
	*sir2*Δ/*sir2*Δ::*SIR2*	*MTL***a**/*MTL*α	0.00±0.00
		*MTL***a**/*MTL*αΔ::FRT	0.00±0.00
		*MTL***a**Δ::FRT/*MTL*α	1.47±1.10
SC5314 *LEU2*/*leu2*Δ *ura3*Δ/Δ *arg4*Δ/Δ *his1*Δ/Δ	*SIR2*/*SIR2*	*MTL***a**/*MTL*αΔ::FRT	1.49±1.20
		*MTL***a**Δ::FRT/*MTL*α	3.11±4.05
	*sir2*Δ/*sir2*Δ	*MTL***a**/*MTL*αΔ::FRT	3.02±2.49
		*MTL***a**Δ::FRT/*MTL*α	3.07±2.04
and *iro1*Δ/Δ	*sir2*Δ/*sir2*Δ	*MTL***a**/*MTL*αΔ::FRT	0.69±0.91
		*MTL***a**Δ::FRT/*MTL*α	0.62±0.69
and 3′-*iro1*Δ/Δ	*sir2*Δ/*sir2*Δ	*MTL***a**/*MTL*αΔ::FRT	1.52±1.16
		*MTL***a**Δ::FRT/*MTL*α	1.49±1.45
BWP17 *LEU2*/*leu2*Δ	*SIR2*/*SIR2*	*MTL***a**/*MTL*α	0.00±0.00
		*MTL***a**/*MTL*αΔ::FRT	0.14±0.39
	*sir2*Δ/*sir2*Δ	*MTL***a**/*MTL*α	0.00±0.00
		*MTL***a**/*MTL*αΔ::FRT	2.70±1.30
		*MTL***a**Δ::FRT/*MTL*α	2.51±0.71

a. ∗FRT denotes that the *SAT1* flipper system was excised and only an FRT site remains.

b.†±indicates, standard deviation.

*SIR2* was able to partially complement the increased W/O switching in the original BWP17 *sir2*Δ/Δ mutants. Complementation of the original BWP17 *sir2*Δ/Δ mutants with an intact copy of *SIR2* reduced the elevated phenotypic switching in both *MTL* genotypes, supporting a specific role for *SIR2* in defining cell state in the original BWP17 *SIR2* strain set [Fig. 1c, Dunn’s post-hoc test (*MTL***a**, WT – *SIR2* complement, *P*=0.0165)]. Addition of an intact copy of *SIR2* to SC5314 *sir2*Δ/Δ mutants or the original BWP17 *MTL***a**/α *sir2*Δ/Δ mutants had no effect on switching (Fig. 1c). Thus, increased white–opaque conversion caused by loss of *SIR2* was specific to the original BWP17 *SIR2* strain set and required *MTL* homozygosity.

### Strain-specific *SIR2* function and slow growth does not explain increased switching

**Fig. 2.**
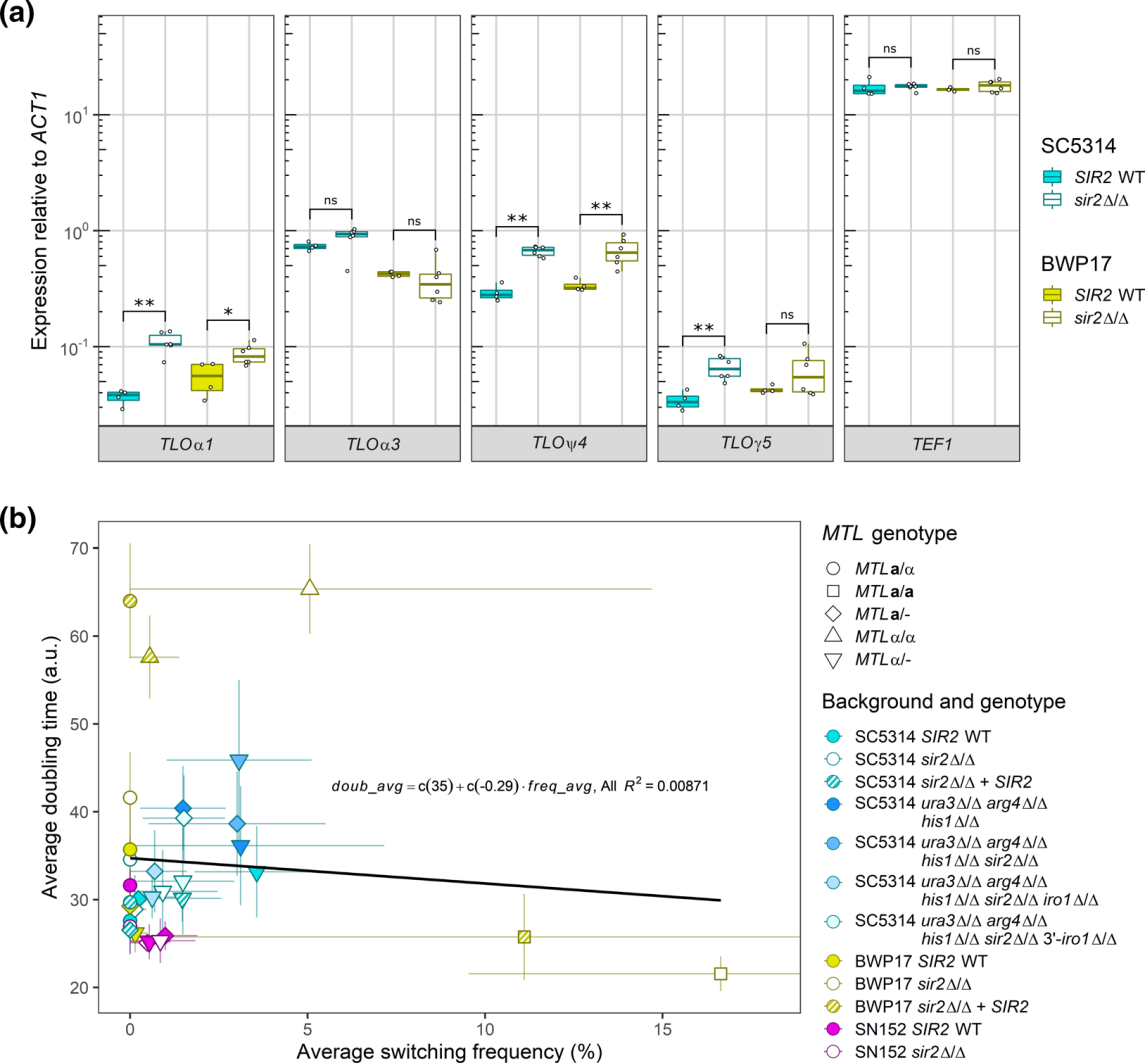
No evidence exists for *altered SIR2* function or slow growth in the original BWP17 *sir2*Δ/Δ mutants. (**a**) Transcript abundance of four subtelomeric *TLO* genes and the non-subtelomeric gene *TEF1* in the original BWP17 and SC5314 *SIR2* wild-type and *sir2*Δ/Δ mutants was measured by qRT-PCR. Overnight cultures grown in liquid YPD medium at 30 °C were diluted into fresh liquid YPD medium and grown for 3–4 h at 30 °C prior to harvesting RNA. Expression was normalized to housekeeping gene *ACT1* with a minimum of four biological replicates. *, *P*<0.05 (Mann–Whitney U test); **, *P*<0.01 (Mann–Whitney U test). (**b**) The doubling time of each strain was calculated in SCD liquid medium during logarithmic phase growth at room temperature. The doubling time and white–opaque switching frequency of each strain is plotted with whiskers indicating the standard deviation. *n*≥4 biological replicates. Each shape denotes the *MTL* configuration. The best fit line for all data points is given in black. For both panels, yellow denotes BWP17-derived strains, magenta denotes SN152 strains and cyan denotes SC5314-derived strains.

Inconsistency between the function of *SIR2* in phenotypic switching between the original BWP17 *SIR2* strain set and other SC5314 lineages led us to investigate what attributes may contribute to increased switching in these strains. *SIR2* was previously implicated in *C. albicans* subtelomeric silencing in SC5314-derived strains [72,73]. Consistent with those previous reports, *SIR2* disruption generally increased expression of the subtelomeric telomere-associated (*TLO*) genes in both the SC5314 and BWP17 genetic backgrounds (Fig. 2a). Of the four subtelomeric *TLO* genes assayed in *sir2*Δ/Δ strains, *TLO*α*1* and *TLO*ψ*4* significantly increased expression in the original BWP17 *sir2*Δ/Δ and SC5314 *sir2*Δ/Δ backgrounds compared to *SIR2* wild-type cells, and *TLO*γ*5* expression increased only in the SC5314 *sir2*Δ/Δ strain [Fig. 2a, Mann–Whitney U test against wild-type: SC5314 (*TLO*α*1*, W=24, *P*=0.0095; *TLO*ψ*4*, W=24, *P*=0.0095; *TLO*γ*5*, W=24, *P*=0.0095); BWP17 (*TLO*α*1*, W=22, *P*=0.0381; *TLO*ψ*4*, W=24, *P*=0.0095)]. Gene expression of *TEF1,* a chromosome internal control gene, was not significantly affected by loss of *SIR2*. Consistent changes in subtelomeric gene expression in *sir2*Δ/Δ mutants from both the BWP17 and SC5314 backgrounds suggested that differences in W/O switching between these backgrounds were not due to altered Sir2-mediated regulation of subtelomeric silencing.

*C. albicans* cells treated with genotoxic stressors or carrying mutations in *RAD51* or *RAD52* grow more slowly and exhibit high rates of W/O switching [61]. To determine whether slow growth of the original BWP17 *sir2*Δ/Δ mutants contributed to increased switching to the opaque state, we calculated the doubling time of wild-type, *sir2*Δ/Δ mutants and their corresponding *SIR2*-complemented mutants across strain backgrounds in liquid SCD at 25 °C (the same conditions used for white–opaque switching on agar medium). The original BWP17 *sir2*Δ/Δ mutants showed opposing growth phenotypes based on the *MTL* genotype despite both having increased W/O switching. The original BWP17 *MTL*α/α *sir2*Δ/Δ strain grew slowly and continued to exhibit slow growth following complementation with an intact *SIR2* (Figs 2b, S1 and S2), whereas the original *MTL***a**/**a** *sir2*Δ/Δ BWP17 strain grew faster than the wild-type parental strain. Thus, the frequency of W/O switching did not correlate with growth rates and is unlikely to account for the elevated switching in the original BWP17 *sir2*Δ/Δ mutants.

### The original BWP17 *sir2*Δ/Δ mutants acquired imbalanced karyotypes

**Fig. 3.**
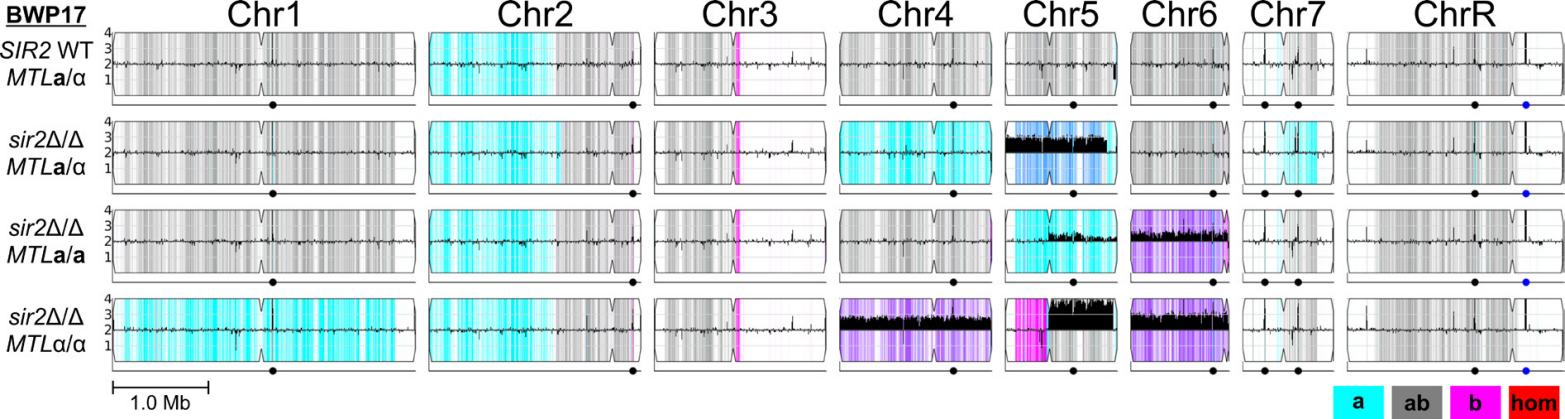
The original BWP17 *sir2*Δ/Δ mutants exhibit complex karyotypes. Whole-genome sequencing of each indicated strain was performed to an average depth of 150× and visualized using Y_MAP_ against Assembly 21 [70]. The height of the solid black bars indicates copy number in 10 kb bins (flat black line=2 N). Grey, cyan and magenta colours represent heterozygous, homozygous homologue A and homozygous homologue B regions, respectively. Blue indicates A/A/B allelic balance and purple indicates A/B/B allelic balance. Red indicates homozygous regions not matching either homologue.

The specificity of increased white–opaque switching to the original BWP17 *sir2*Δ/Δ mutants suggested that unique evolutionary events may have occurred in this lineage. To identify genotypic changes in the original BWP17 *sir2*Δ/Δ strains that displayed increased W/O phenotypic switching, whole-genome sequencing was performed for the original BWP17 *SIR2* wild-type background strain and the corresponding original BWP17 *sir2*Δ/Δ mutants in all three *MTL* configurations (*MTL***a**/α, *MTL***a**/**a** and *MTL*α/α). The genome of the original BWP17 *SIR2* wild-type background strain was similar to published reports with no new major LOH or CNV events (Fig. 3). However, construction of the original BWP17 *sir2*Δ/Δ mutants in the *MTL***a**/α background introduced three major karyotypic changes: whole chromosome LOH events for Chr4 and Ch7 and acquisition of a Chr5 trisomy (AAB genotype).

Production of the original BWP17 *MTL***a**/**a** and *MTL*α/α *sir2*Δ/Δ mutants resulted in additional genomic changes unique to each *MTL* homozygous strain. The original BWP17 *MTL***a**/**a** *sir2*Δ/Δ mutant retained heterozygosity of Chr4 and Chr7, indicating an original mixed population in the *MTL* heterozygote or more recent LOH events for these chromosomes in the *MTL***a**/α background (Fig. 3). The original BWP17 *MTL***a**/**a** *sir2*Δ/Δ mutant also appeared to be a mixed population of cells disomic or trisomic for the right arm of Chr5 (Chr5R), although all cells had undergone whole chromosome LOH (AA or AAA genotype). Chr6 in the original BWP17 *MTL***a**/**a** *sir2*Δ/Δ mutant was also trisomic (ABB configuration) and subsequently underwent LOH of Chr6R to acquire a BBB genotype. Complementation of the original BWP17 *MTL***a**/**a** *sir2*Δ/Δ mutant did not significantly alter this complex karyotype but indicated that the *MTL***a**/**a** *sir2*Δ/Δ mutant was composed of a mixed population of diploid and Chr6 trisomic/Chr5R tetrasomic cells (Fig. S3).

The original BWP17 *MTL*α/α *sir2*Δ/Δ mutant contained even more complex genomic changes than its *MTL***a**/**a** counterpart. Chr1 underwent whole-chromosome LOH, and both Chr4 and Chr7 were present as ABB trisomies (Fig. 3). The left arm of Chr5 appeared to have homozygosed the B homologue, although the right arm was heterozygous and present in four copies. The most parsimonious explanation for this genotype would be the formation of an isochromosome of Chr5R for the A homologue along with the presence of two full homologue B chromosomes (Fig. 3). The *SIR2*-complemented BWP17 *MTL*α/α *sir2*Δ/Δ mutant carried an indistinguishable karyotype to the mutant (Fig. S3). Thus, the only karyotypic change in common between both original BWP17 *MTL* homozygous *sir2*Δ/Δ mutants is the ABB Chr6 trisomy, although it is unclear whether this would be sufficient to increase opaque formation in *MTL* homozygous BWP17 strains.

Sequencing of SC5314 strains constructed to test the function of *SIR2* in W/O switching did not undergo significant genomic rearrangements. Unexpectedly, one of the two CRISPR-competent SC5314 parental strains used to construct the *SIR2* mutants, MAY1035, was trisomic for Chr5 (ABB), while the other, MAY1244, was fully diploid (Fig. S3). MAY1035 was used to construct the CRISPR *SIR2 MTL***a**/α and *MTL*α/− sets, while MAY1244 was used to construct the *MTL***a**/− set. All *SIR2* strains constructed using CRISPR in this SC5314 background retained their parental ploidies. A handful of strains, the *MTL***a**/α *sir2*Δ/Δ and *MTL***a**/− *sir2*Δ/Δ sets, possessed LOH events at or proximal to the *SIR2* locus that are likely products of break induced replication (BIR) mechanisms of DNA repair caused by CRISPR-induced DNA breaks (Fig. S3). Therefore, Chr5 trisomy has no impact on W/O switching and further argues for a potential role for Chr6 trisomy to interact with *sir2*Δ/Δ in the original BWP17 strain set.

## Discussion

Here, we sought to determine the role of *SIR2* in phenotypic switching based on preliminary observations that appeared to support a role for *SIR2* regulation of W/O switching. Analysis of a large set of *sir2*Δ/Δ mutants produced in multiple SC5314-derived backgrounds clearly demonstrated that increased W/O switching was restricted to the original BWP17 *SIR2* strain set including these mutants. Loss of *SIR2* did not alter phenotypic switching in any other SC5314-derived strains, including CRISPR-competent BWP17 *MTL***a**/− and *MTL*α/− *sir2*Δ/Δ mutants. Increased white–opaque switching in these *sir2*Δ/Δ mutants was also not a consequence of slow growth, and *SIR2* retained its canonical function in regulation of subtelomeric gene expression in the original BWP17 background. Instead, increased phenotypic switching in the original BWP17 *sir2*Δ/Δ strains may be linked to large-scale karyotypic changes that can alter regulation of the encoded genes. Therefore, *SIR2* does not regulate white–opaque switching in the SC5314 background on its own but may be able to interact with other unknown genetic factors introduced during strain construction to dysregulate cell state and increase opaque cell formation.

The importance of characterizing the genetic background of strains to accurately interpret research findings in *C. albicans* is prominently evident in these results. Both our preliminary data and prior work pointed to *SIR2* repressing the transition to the opaque cell state [58]. However, the inability to replicate these findings in four different SC5314 lineages, two of which contained the same targeted genetic changes, demonstrated that the *SIR2* phenotype was attributable to the original BWP17 *sir2*Δ/Δ strain set’s genetic background and not an intentional aspect of strain construction. Our results instead support a prior characterization of genes encoding selected chromatin-modifying enzymes in the white–opaque switch that failed to find any roles for *SIR2* using an SN lineage strain background [55]. It is possible that the original reports of increased phenotypic switching caused by inactivation of *SIR2* may have been complicated by karyotypic changes found in those *sir2*Δ/Δ strains, as in our original BWP17 *sir2*Δ/Δ mutants. The authors noted the consistent presence of an extrachromosomal band in *sir2*Δ/Δ mutants, which indicates the potential for karyotypic alterations or instability to interact with phenotypic switching [58]. However, *SIR2* does possess a function in cell state transition, as it has recently been found to promote the yeast to hyphal transition [74].

Another major point raised by this study is the importance of using multiple *C. albicans* lineages and, ideally, backgrounds in assessing mutant phenotypes. First, we strongly recommend that construction of *C. albicans* strains be performed in a ‘clean’ genetic background as frequently as possible. Strain construction is now easily accomplished in less manipulated, prototrophic backgrounds through a myriad of available resources that function across genetic backgrounds [60,75, 76]. Second, multiple independent lineages should be built during all strain construction. This is not the case with the BWP17 and SN lineages or the original BWP17 *sir2*Δ/Δ mutants. The potential for mutations to arise during laboratory manipulations is well documented and strongly argues for the sequencing of important laboratory strains following construction. It is also no longer necessary to work in a single strain background with *C. albicans*. A growing compendium of sequenced isolates available for laboratory use and their associated phenotype data provides a solid platform for identifying natural variants in genes of interest for investigation or interpreting results in the genome reference strain [77,78]. Use of multiple strains can refine the centrality of genes to *C. albicans* biological processes and the likelihood of clinical applicability, as not all mutations behave similarly across strain backgrounds [4].

Increased opaque cell formation in the original BWP17 *sir2*Δ/Δ mutants was associated with large-scale chromosomal changes in the mutant background. BWP17 contains multiple karyotypic changes relative to SC5314 that accumulated during its construction [21]. The presence of additional LOH tracts and imbalanced chromosomes exacerbated these genotypic differences in the original BWP17 *MTL***a**/α *sir2*Δ/Δ mutant and its *MTL* homozygous counterparts. The only karyotypic change in common for both the high switching original BWP17 *MTL***a**/**a** and *MTL*α/α *sir2*Δ/Δ mutants is a trisomic Chr6 with an ABB haplotype. High-frequency switching *sir2*Δ/Δ mutants in the original BWP17 *SIR2* strain set also harbour at least a Chr5R tetrasomic subpopulation that is probably present as an isochromosome of two Chr5R arms. We favour a potential role for Chr6 in promoting opaque cell formation as increased dosage of Chr5 in trisomic lineages of the SC5314 CRISPR strain set did not alter W/O switching. None of the canonical chromatin modifiers or transcription factors that promote the opaque state are present on Chr6 or Chr5R, so it is unclear how dosage could be contributing to increased switching. Indeed, prior work did not find any association between ploidy or aneuploidy changes and cell state [79], and systematic phenotyping of trisomic strains in SC5314 failed to note any effects on white–opaque switching, although these studies were performed in *MTL***a**/α cells [32].

Increased white–opaque switching in the original BWP17 *sir2*Δ/Δ mutants suggests that *SIR2* represses formation of the opaque state in this lineage. Deacetylation activity of histones by Sir2 is limited to subtelomeric regions and the rDNA locus [80]. Increased dosage of genes on amplified chromosomes in combination with loss of subtelomeric silencing may provide sufficient expression of some regulator of cell state that increases opaque cell formation.

The potential genetic interaction between trisomic chromosomes, LOH and loss-of-function mutations that seems to underlie this study highlights the lack of understanding of complex regulatory mechanisms in *C. albicans*. Major gaps in our understanding of the molecular and biological functions of *SIR2* and other chromatin modifiers in *C. albicans* complicate linking the *SIR2*-dependent switching phenotype to any specific genotypic aspect of the original BWP17 *sir2*Δ/Δ strains. Many of the previously described functions of *SIR2* in *C. albicans* are conserved with *SIR2* in *Saccharomyces cerevisiae*: (1) H3K9ac and H4K16ac targets, (2) subtelomeric and rDNA silencing and (3) altered cell longevity [72,80,82]. Yet, *C. albicans SIR2* also has additional functions that have not been reported in *S. cerevisiae*, such as suppressing subtelomeric recombination and gene noise [72,83]. Importantly, no studies have investigated roles for *C. albicans* sirtuins, including *SIR2*, in removal of other acyl groups or modification of non-histone proteins [84]. How loss of *SIR2* in a genotypically complex background could lead to altered cell states may suggest additional roles in regulating expression through dosage or allele-specific expression that have not been investigated [85]. More mechanistic understanding of how copy number and allelic representation alter molecular functions is needed to connect how mutations may alter cell physiology.

## supplementary material

10.1099/mic.0.001444 Uncited Fig. S1.

10.1099/mic.0.001444 Uncited Table S1.

10.1099/mic.0.001444 Uncited Table S2.

10.1099/mic.0.001444 Uncited Table S3.

## Abbreviations

AMSL: Applied Microbiology Services Laboratory
ARG: arginine
bp: basepairs
Chrx[L/R]: chromosome #, left/right arm
CNV: copy number variation
dDNA: donor DNA
gRNA: guide RNA
HIS: histidine
kb: kilobases
LEU: leucine
LOH: loss of heterozygosity
*MTL*: mating type-like
NAT: nourseothricin
NCBI: National Center for Biotechnology Information
OD: optical density
oligos: oligonucleotides
ORF: open reading frame
PBS: phosphate-buffered saline
PCR: polymerase chain reaction
qRT-PCR: quantitative reverse transcription PCR
SCD: synthetic complete dextrose
URA: uracil/uridine
W/O: white-opaque
YPD: yeast extract-peptone-dextrose
YPM: yeast extract-peptone-maltose
