# Supplementary material for: Strain background of Candida albicans interacts with SIR2 to alter phenotypic switching
Source: Microbiology (Reading). 2024 Mar 6;170(3):001444. doi: 10.1099/mic.0.001444 (PMC10999749; doi:10.1099/mic.0.001444)
Supplement: Uncited Fig. S1. [file mic-170-01444-s001.pdf]

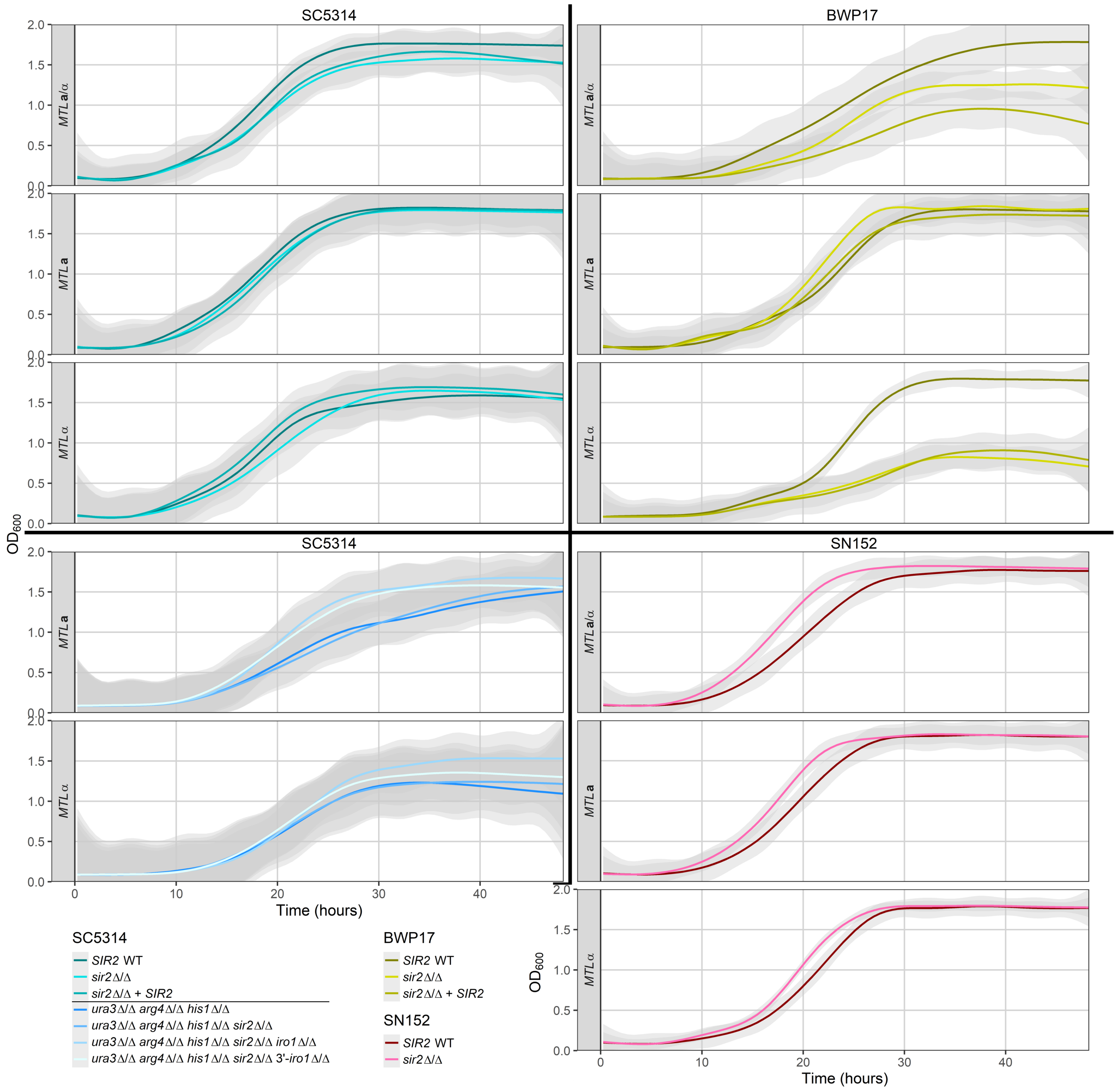

Figure S1. Growth curves of the SIR2 strain sets. Growth curves for strains in SCD liquid medium at room temperature with the line indicating the average and standard deviations as grey shading. N ≥ 4 biological replicates. Cyan/blue denotes SC5314-derived strains, yellow denotes the original BWP17-derived strains, and magenta denotes SN152 strains.

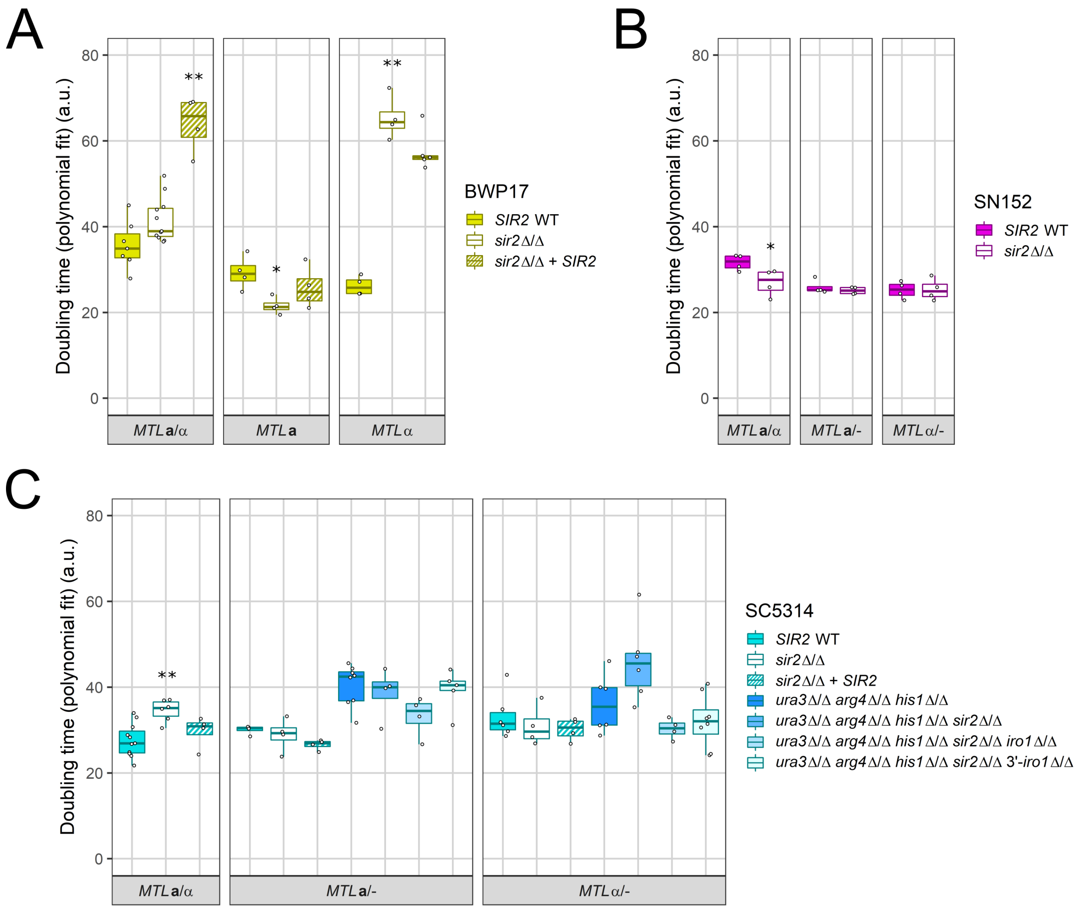

Figure S2. Growth rates of the SIR2 strain sets. The doubling time in logarithmic phase growth was calculated for strains in SCD liquid medium at room temperature and plotted as the mean with standard deviations. N ≥ 4 biological replicates. (A) Yellow denotes the original BWP17-derived strains, (B) magenta denotes SN152 strains, and (C) cyan denotes SC5314-derived strains. *, P < 0.05 (Kruskal-Wallis test with Dunn's post-hoc against wildtype). **, P < 0.01 (Kruskal-Wallis test with Dunn's post-hoc against wildtype). Wildtype = WT.

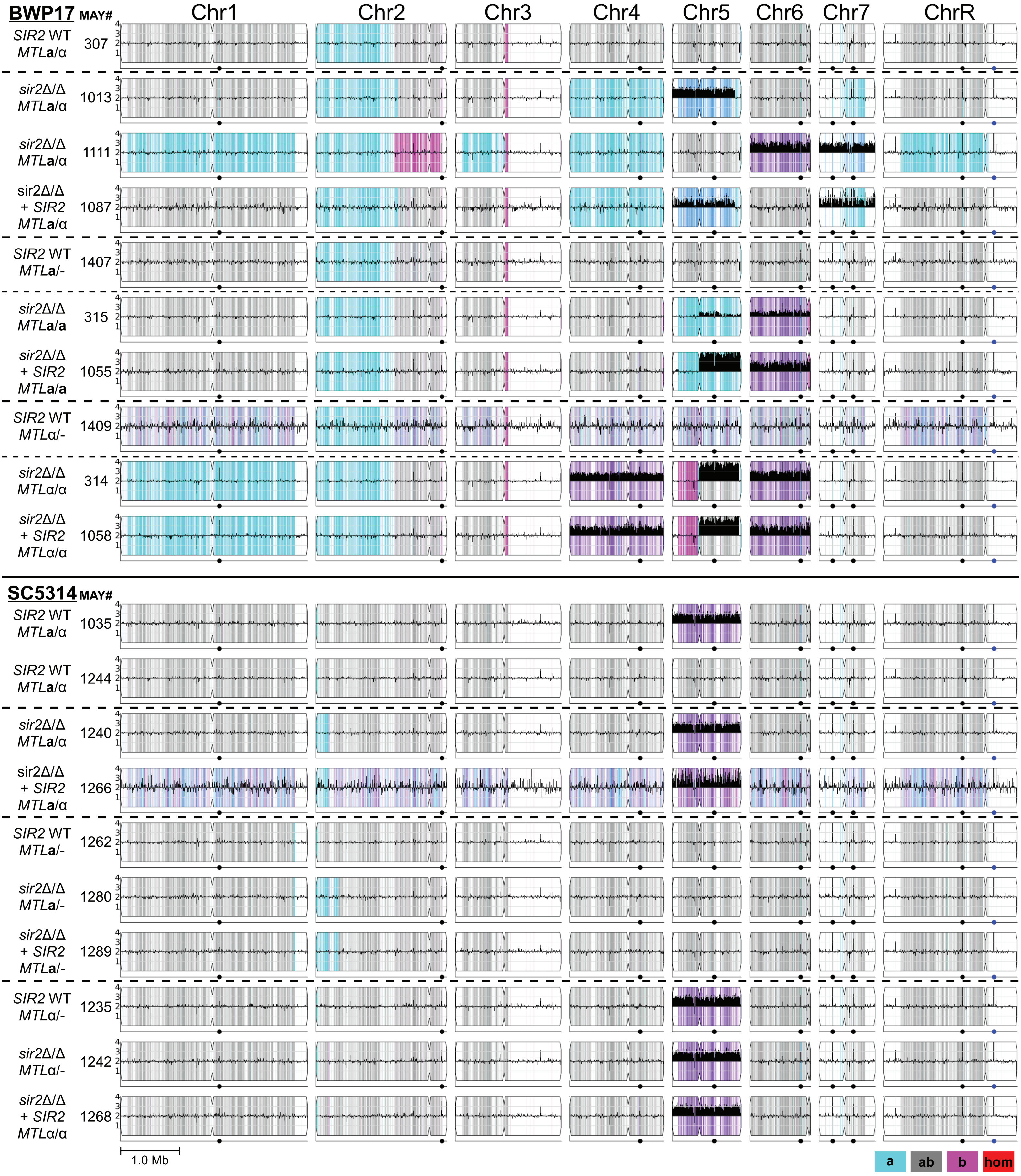

Figure S3. Whole genome sequencing and visualization of the original BWP17 SIR2 and CRISPR SIR2 strain sets. Whole genome sequencing of each indicated strain was performed to an average depth of 26.8X and visualized using YMAP against Assembly 21 [71]. MAY# indicates the strain identifier of the sequenced strain. The height of the solid black bars indicates copy number in 10 kilobase bins (flat black line = 2N). Grey, cyan, and magenta colors represent heterozygous, homozygous homolog A, and homozygous homolog B regions respectively. Blue indicates A/A/B allelic balance, and purple indicates A/B/B allelic balance. Red indicates homozygous regions not matching either homolog.
